# Therapeutic Targeting of the Tumour Microenvironment in Metastatic Colorectal Cancer

**DOI:** 10.3390/ijms22042067

**Published:** 2021-02-19

**Authors:** Rhynelle S. Dmello, Sarah Q. To, Ashwini L. Chand

**Affiliations:** Cancer and Inflammation Program, Olivia Newton-John Cancer Research Institute and School of Cancer Medicine, La Trobe University, Heidelberg, VIC 3084, Australia; Rhynelle.dmello@onjcri.org.au (R.S.D.); Sarah.To@onjcri.org.au (S.Q.T.)

**Keywords:** metastatic colorectal cancer, targeted therapy, immunotherapy, tumour microenvironment, inflammation, cytokines, tumour heterogeneity

## Abstract

Liver metastasis is the primary contributor to the death of patients with colorectal cancer. Despite the overall success of current treatments including targeted therapy, chemotherapy, and immunotherapy combinations in colorectal cancer patients, the prognosis of patients with liver metastasis remains poor. Recent studies have highlighted the importance of the tumour microenvironment and the crosstalk within that determines the fate of circulating tumour cells in distant organs. Understanding the interactions between liver resident cells and tumour cells colonising the liver opens new therapeutic windows for the successful treatment of metastatic colorectal cancer. Here we discuss critical cellular interactions within the tumour microenvironment in primary tumours and in liver metastases that highlight potential therapeutic targets. We also discuss recent therapeutic advances for the treatment of metastatic colorectal cancer.

## 1. Introduction

### 1.1. Incidence

Colorectal cancer is the third most commonly occurring cancer in men and the second most commonly occurring cancer in women. The World Health Organisation estimates on worldwide colon cancer incidence indicate an increase from ~1 million to 1.5 million new cases by 2030. Additionally, there is a predicted increase in colon cancer-related mortality from ~500,000 (data in 2018) to 750,000 deaths by 2030 [1,2]. Current data indicates that the cumulative lifetime risk of colon cancer in men is 1.51% and 1.12% in women [3,4]. This correlates to ~576,000 and ~521,000 colon cancer diagnoses in men and women, respectively. Colon cancer risk is significantly greater in populations in certain countries in Europe, and in countries such as USA, Australia, and New Zealand [3,4,5]. Incidence data is correlated highly with dietary and lifestyle factors including alcohol consumption, smoking, diet and BMI, physical activity, comorbidities including obesity, as well as the use of medications such as non-steroidal anti-inflammatory drugs [4,6]. High incidence rates recorded are likely to reflect higher screening rates with increased patient monitoring and the availability of improved screening tools. Genetic predisposition, as well as disparity in access to quality healthcare, screening/early detection, and socioeconomic factors, are attributed to different rates of colon cancer incidence and mortality rates. Interestingly, while incidence rates have dropped by ~3.6% each year from 2007 to 2016 in older adults greater than 55 years in age, incidence rates have risen by 2% each year in younger adults (20–49 years), a trend observed worldwide [4,5,7]. This alarming trend in the increase in colon cancer in the younger demographic is suggestive of lifestyle and environmental factors as contributors to early onset. In addition, the current global pandemic of coronavirus disease 2019 (COVID-19), caused by severe acute respiratory syndrome coronavirus 2 (SARS-CoV-2), will influence rates of detection rates, accessibility to treatment, as well as changes in approaches employed in the management of colorectal cancer during the period of 2020 and 2021 [8,9,10]. These changing circumstances worldwide are likely, in future, to have significant influence on colon cancer mortality and incidence rates in a manner previously not observed.

### 1.2. Prognosis

Global predictions of colon cancer mortality rates indicate that in 2020, there was a ~5% increase when compared to 2018 in all ages [1,3,4,5]. In the United States, the five-year survival rate for individuals with all stages of colorectal cancer is estimated at ~64% [2]. Dependent on the stage of cancer, the five-year survival rates are greatly varied, with localised colorectal cancer having an expected survival rate of 90%. In patients with invasive cancer into surrounding tissue and lymph nodes, survival rates are 70%, and with advanced stage disease where distal metastasis has occurred, survival rates are drastically reduced to 14% [2,11]. Metastasis to distant parts of the body includes to the liver, lungs, and distant lymph nodes. Approximately 20–25% of patients with synchronous cancer present with metastases at time of diagnosis with a poor five-year survival rate of ~10% compared with those with localised disease. Similar rates ~20% of metachronous metastases are observed, with a majority (83%) of these metastases diagnosed within three years, with the most metastasis to the liver [12]. Moreover, advanced staged primary tumour status and tumours identified in the descending colon or the rectum are positively correlated to metastatic disease [12]. Hence, a high incidence of liver metastases occurs in ~25% of all patients with colorectal cancer, and currently there are no prognostic patterns discriminating between synchronous versus metachronous detection of metastases [13]. 

Metastatic disease can be controlled with systemic treatments, chemotherapy being the mainstay for treatment, with its efficacy bolstered by combination with targeted therapies including cetuximab, panitumumab, bevacizumab, binimetinib, ramucirumab, donafenib, and regorafenib. Development of new targeted treatments is geared towards the improvement of progression-free survival as well as the management of side-effects associated with chemotherapy including neutropenia, diarrhoea, vomiting and nausea, hepatotoxicity, and vascular injury [14,15]. Approaches of reducing treatment duration and alleviating side-effects associated with prolonged use of chemotherapy include the evaluation of efficacy of various treatment regimens including sequential vs. combination and continuous vs. intermittent use of chemotherapy regimens and progress our understanding of improved parameters necessary for the management of patients with metastatic colorectal cancer [14,16,17,18]. 

## 2. Colon Cancer Subtypes

### 2.1. Molecular Classification of Colon Cancer 

In addition to understanding the genomic landscape of the tumour cells, the interplay of stromal and immune cell components of the tumour microenvironment influences the best approaches towards individualised treatment [19,20]. Commonly identified driver mutations associated with colorectal cancer include those in tumour suppressor genes such as adenomatous polyposis coli (*APC*), *TP53*, and SMAD family member 4 (*SMAD4*) and oncogenes including *KRAS* and PI3K catalytic subunit-α (*PIK3CA*). Recently, with gene expression profiling data, four consensus molecular subtypes (CMS) have been structured (Table 1). Tumours characterised with microsatellite instability (MSI) associated with hyper mutation and hyper methylation patterns, an enrichment for *BRAF*V600E mutations, and a high immune cell infiltration (CD8^+^ cytotoxic T lymphocytes, CD4^+^ T helper 1 (TH1) cells and natural killer (NK) cells) in the tumour microenvironment are classed within CMS1 [21,22]. Defects in the DNA mismatch repair pathway in CMS1 also display upregulated expression of multiple immune checkpoints including CTL-associated antigen 4 (CTLA4), programmed cell death protein 1 (PD1), PD1 ligand 1 (PDL1), and indole amine 2,3-dioxygenase 1 (IDO1) [22]. Given that tumour infiltrating immune cells are found in tumours with high mutational burden, the expression of immunogenic epitopes on tumour epithelial cells accounts for a strong TH1 immune response, formation of tertiary lymphoid structures, and the activation of the Janus kinase (JAK)-signal transducer and activator of transcription (STAT)-dependent immune signalling pathways [19,20,23].

Tumours with chromosomal instability (CIN) are subclassified into three groups, CMS2-4. The CMS2 subtype represents 37% of early-stage tumours, characterised by the upregulation of WNT and MYC downstream target gene signatures, higher expression of *EGFR*, *ERBB2* (HER2), insulin-like growth factor 2 (*IGF2*), insulin receptor substrate 2 (*IRS2*), and transcription factor hepatocyte nuclear factor 4α (*HNF4A*) [19]. The CMS3 subtype encompasses tumours displaying a high metabolic phenotype associated with activation of glutaminolysis and lipogenesis, with distinctive genomic and epigenomic profiles and with consistently fewer copy number alterations. This subtype also contains MSI tumours, hyper mutation, and intermediate levels of gene hyper methylation in ~30% of the CMS3 subtype. Tumours in the CMS3 subtype are enriched for *KRAS*-activating mutations, which have been linked to distinct metabolic pathways [19]. The fourth subtype, CMS4, accounts for mesenchymal tumours and activated stromal fibroblast features. The gene expression signature for the tumours classified as CMS4 subtype is strongly associated with transforming growth factor-β (TGFβ) activation from a stromal-enriched inflamed microenvironment. It is also associated with gene signatures linked with epithelial–mesenchymal transition (EMT) and extracellular matrix remodelling. The molecular classification of colorectal cancers is paving the way to determining the responsiveness of different therapeutic responses in each subtype, as well as identifying new molecular targets that can be used for drug discovery efforts geared to ultimately designing specific treatment of these distinct tumour subtypes. 

### 2.2. Understanding Heterogeneity in Metastatic Colorectal Cancer

In metastatic disease, genomic mutational signatures and gene expression profiles vary from those identified in the primary tumour. Furthermore, the analysis of immune cell types within the tumour microenvironment of metastases and primary colorectal tumours show a heterogeneous immune infiltrate profile (also termed Immunoscore) as well as varied mutational status [21,26]. The interrelationship between the extent of genetic mutational burden correlates with distinct immune-infiltration patterns within the tumours and both factors collectively affect patient treatment responses and overall survival outcomes. A significant study by Galon and colleagues demonstrated that tumour immunoscore is heterogeneous between metastases from the same patient [26]. Moreover, metastases with lowest immune infiltration were a prognostic factor for tumour relapse and decreased survival outcomes [26]. Interestingly, treatment with EGFR antibodies altered immune-related gene signatures reflective of increased T cell densities within the metastases [26]. This indicates that the responses to various targeted treatments and the resulting changes in the tumour microenvironment would provide further insights into combination treatments with immune-checkpoint inhibitors in metastatic colorectal cancer. Additionally, the prognostic role of tumour immunoscores (CD3, CD8, granzyme B, or FOXP3^+^ immune cells) in metastatic colorectal cancer lesions at the invasive margin of liver metastases correlated positively to chemotherapy responses with 100% specificity, with high immune infiltrates correlating with longer progression-free survival in patients who received chemotherapy [27]. 

## 3. Colorectal Cancer and Its Tumour Microenvironment 

### 3.1. Chronic Inflammation and the Primary Tumour Microenvironment 

The integrated cellular processes that contribute to the development and progression of cancer are now defined as the hallmarks of cancer including genomic instability, uncontrolled cell proliferation, evasion of cellular apoptosis, stromal remodeling, and inflammation [28,29]. 

The role of inflammation in cancer involves tissue remodeling, eliciting a range of immune responses and promoting cellular plasticity contributing to tumour invasion and metastasis. Inflammation is a homeostatic reparative process associated with tissue injury and infection, and dysregulated, chronic inflammation leads to the disruption of tissue homeostasis, providing a pro-tumourigenic environment [30]. Chronic intestinal inflammation is a known risk factor for the development of colorectal cancers [31]. The primary tumour microenvironment in colorectal cancer comprises of a lumen that is microbiome-rich, and a barrier surface that is dynamic in its tissue remodeling and regenerative processes. The presence of a tumour is also influenced by adaptive immune responses and a strong emphasis on stem cell and tissue regeneration processes, which are associated with tumour initiation and development in the colon. In this context, in colorectal cancer, there is a diverse range of tumour microenvironments that support neoplastic transformation and metastatic dissemination of cancer cells. Moreover, these complex interactions between the tumour and its microenvironment influence therapeutic efficacy of standard of care chemotherapy and emerging targeted therapies as well as checkpoint inhibitors. 

Cancer-associated stromal fibroblasts play a key role in the development of colon carcinomas, and their TGFβ-dependent reprogramming of cancer-associated fibroblasts (CAFs) is significantly correlated with relapse and metastatic disease [32,33]. Together with the fact that mutational inactivation of TGFβ is frequently observed, combined with elegant in vivo mechanistic studies, it has been shown that TGFβ is a key driver in altering CAF features to increase the efficiency of metastasis to the liver [32]. The key downstream activator of crosstalk between CAFs and the tumour epithelial cells is interleukin 11 (IL-11), upregulated in the CAFs by tumour-derived TGFβ, a key cytokine-dependent mechanism that promotes a prometastatic phenotype in tumour epithelial cells. Further, the colonisation of colorectal cancer cells into the liver is driven through the influence of TGFβ-mediated changes to the liver as the host organ for metastatic growth. 

The recent molecular classification of colorectal cancer subtypes reflects not only on the heterogeneous nature of colorectal cancer within tumour epithelial cells, but also the tumour microenvironment. Tumour cells with a high mutational burden and neoantigen presentation have a greater number of immune cell infiltrates and respond more efficaciously to immune checkpoint inhibitors. Conversely, tumours with low mutational burden display clear exclusion of T lymphocytes [20]. Interestingly, inhibition of TGFβ evoked enhanced T-cell activation and increased responsiveness to immune checkpoint inhibition [33]. 

The immune context within the tumour microenvironment of colorectal cancer has been well characterised, as well as spatio-temporal patterns associated with “tumour-host” responses and patient survival outcomes [34]. The prognostic significance of the Immunoscore, generated by the work of Galon et al., is based on the quantification of CD3^+^ and CD8^+^ T cells at the tumour core and at the invasive margin. The patterns of immune cell infiltrates into the tumours are strongly correlated with MSI status, which also correlates with responses to immune checkpoint blockade [35,36]. Additionally, immune responses in MSI-high tumours and high neoantigen load are correlated with reduced disease severity and metastasis, with <5% of MSI-high patients showing metastatic disease [35,36]. This negative correlation to distant metastasis was due to decreased number of lymphatic vessels and reduced immune cytotoxicity [37]. Further delineation of the complex mechanisms via which the tumour cells reshape its microenvironment is warranted for the design of improved therapeutics for advanced colorectal cancer. 

### 3.2. Cellular Components of the CRC Microenvironment in the Liver

In colorectal cancer, the liver is the most colonised organ for distal metastatic outgrowth, due to its positioning and significant blood supply via the major vascularity (hepatic artery and portal vein) and microvasculature. Branching structures of the vasculature into the liver sinusoids become lodgement sites for tumour cells in the circulation [37]. Despite the liver being a susceptible organ for metastasis, its microenvironment is initially hostile for the invading tumour cells. Strong anti-tumour responses, which are mediated by the liver include the liver sinusoidal endothelial cells (LSECs), evoking pro-apoptotic responses with nitric oxide release and the generation of reactive oxygen species. Apoptosis is also induced by natural killer cells (NK) through the release of perforin and granzymes, while Kupffer cells drive phagocytosis. This hostile microenvironment provides a strong trigger for clonal expansion of tumour cells with diverse adaptive mechanisms, including the evasion of apoptosis via physical lodgement into the hepatic sinusoids and a heightened secretion of cytokines, such as TGFβ, produced by tumour cells to trigger the hepatic stellate cells (HSCs) into a desmoplastic response. These changes provide a scaffold for angiogenesis and the establishment of micro-metastases [38,39]. The presence of micro-metastases lesions activates specific immune responses in the liver, which the tumour cell is able to evade via the upregulation of PD-1 and CTLA-4, causing inhibition of effector T cell function and the metastatic growth acquiring immune tolerance. The varied rates of proliferation and expansion of micro-metastases to macroscopic metastases reflect clonal differences, with some clones proliferating to become larger lesions while some persist as micro-metastases [39,40]. 

Akin to the pro-tumourigenic signalling pathways that regulate primary colorectal cancer progression, within metastatic lesions, the activation of Wnt, TGF-β, BMP, NOTCH, and STAT3 signalling pathways, as well as cellular metabolic and cell survival pathways including PI3K/AKT and MAPK, drive metastatic outgrowth. In the liver, pathways activated by mechanical transduction including members of the HIPPO pathway may have a major effect on the modulation of liver metastasis [41]. The prominence of IL-6 signalling in the liver as well as TGFβ-driven IL-11 signalling in supporting primary tumour growth and metastasis in preclinical models suggest that these cytokines play important roles in the outgrowth of hepatic metastases [32,42]. Taken together, the liver presents a highly complex tumour microenvironment due to the specialised cell types present and their varied responses to pro-tumourigenic signalling pathways. Understanding how these factors influence clonal selection and contribute to heterogeneous immune responses could lead the way to improved treatments targeted specifically at reducing colon cancer metastatic outgrowth in the liver.

## 4. The Role of the Liver Microenvironment in the Formation of a Pre-Metastatic Niche 

### 4.1. The Metastatic Process in Colon Cancer

**Figure 1:**
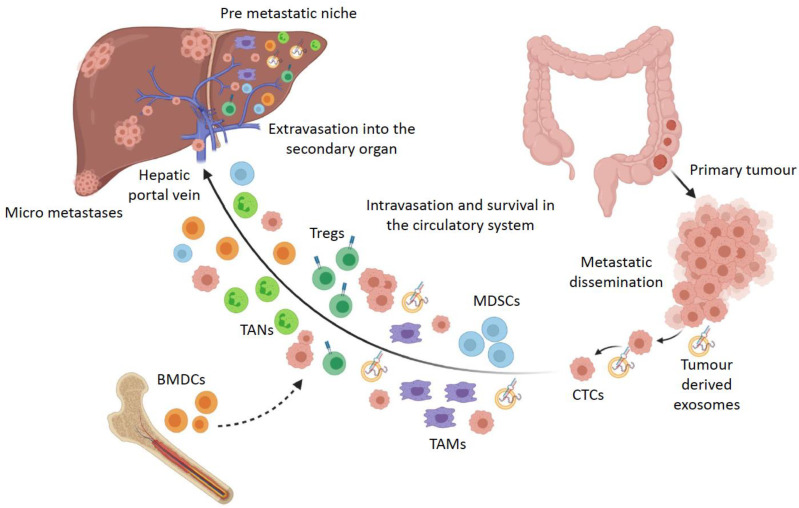
The metastatic cascade in colorectal cancer: fate of circulating tumour cells and components of the pre-metastatic niche. Disseminated cancer cells from the primary tumour travel through the circulation and enter the liver via the hepatic portal vein along with tumour derived factors, bone marrow-derived dendritic cells (BMDCs), and immune cells that prime the liver microenvironment for the colonisation of circulating tumour cells (CTCs) into micro-metastases.

The multi-step process of colon tumourigenesis from a normal colonic epithelium to metastatic dissemination is supported by the tumour microenvironment. The metastatic cascade has been characterised into several stages of local infiltration of primary tumour cells into adjacent tissue, intravasation, survival in the circulatory system, extravasation, and proliferation in the secondary organ (Figure 1) [43,44,45]. Particularly in colon cancer progression, cells undergo EMT along with the loss of cell adhesion capacity, which allows the dissemination of malignant cells from the carcinoma that intravasate into lymph nodes and blood vessels through a fragmented basement membrane [46,47]. Cells acquire invasive properties as a result of chromosomal instability induced by genetic and epigenetic factors, ECM components providing a structural framework for cell migration, soluble signals from growth factors and cytokines, and mechanical pressure generated by the ECM [48]. Clusters of migrating carcinoma cells that are a hybrid of epithelial and mesenchymal phenotypes are more likely to survive in the circulatory system and therefore colonise secondary organs and form micro-metastases [46,49]. 

Tumour cells in different stages of EMT are localised in different microenvironments and interact with diverse subpopulations of stromal cells, contributing to their heterogeneity. In colon cancer cells undergoing metastasis, the expression of transcription factors such as SNAIL, TWIST, ZEB, and others are upregulated, which plays an important role in suppressing the epithelial phenotype and activating the mesenchymal phenotype [50]. Cells that are closer to the mesenchymal phenotype secrete chemokines and growth factors such as prostaglandin E2 (PGE2), macrophage-colony stimulating factor (M-CSF), and interleukin 6 (IL-6) that recruit immune cells and promote angiogenesis, thereby forming an inflammatory, vascularised niche [49,51,52]. Out of these, IL-6 is highly expressed in colon cancer cells and promotes progression into liver metastasis through IL-6/JAK/STAT signalling [52,53].

### 4.2. Formation of a Pre-Metastatic Niche in the Liver

In order to successfully colonise distant organs, circulating tumour cells must be able to survive in the circulation, infiltrate into distant tissue, evade immune defence systems, adapt to diverse niche environments, and survive as latent ‘seeds’ that eventually break out and colonise the secondary tissue [40]. The concept that the spread of cancer cells depends on the interaction of the cancer cell (seed) and the host organ (soil) has been studied extensively since the proposal of this theory and has resulted in the establishment of the notion that specific organs are predisposed to metastases in specific cancers, and that the cross talk between cytokine and chemokine signalling and tumour cells regulates metastatic colonisation in secondary organs [54]. The term ‘pre-metastatic niche’ refers to the microenvironment in the secondary organ that supports the colonisation of metastatic tumour cells by providing favourable conditions that change in the otherwise hostile environment of the secondary organ (Figure 1) [38,55,56,57]. This environment is created by the recruitment of tumour-associated macrophages (TAMs) and tumour-associated neutrophils (TANs), myeloid derived suppressor cells (MDSCs), and regulatory T cells (Tregs) to the secondary site by cytokines, chemokines, and inflammatory factors from the primary tumour, which ‘prime’ the environment in the secondary organ before the arrival of circulatory tumour cells [38,55,56,57]. The formation of the pre-metastatic niche involves bone marrow-derived haematopoietic progenitor cells (BMDCs) that express VEGFR1 to form cellular clusters, promoting the upregulation of fibronectin in tissue resident fibroblasts to prepare the pre-metastatic site for the arrival of circulating tumour cells [58]. Colon cancer cells secrete VEGF-A, which stimulates CXCL1 expression in the primary tumour by TAMs [59]. Elevated levels of CXCL1 recruit CXCR2 positive MDSCs to the pre-metastatic liver tissue and promote tumour cell survival and metastasis while evading host immune responses [59]. 

Recently, cancer-derived exosomes carrying cargo including cytokines and miRNAs have emerged as key contributors to the preparation of the pre-metastatic niche, mediating cancer-induced vascular permeability, inflammation, and recruitment of bone marrow-derived progenitor cells in distal organs [60,61,62]. In the liver, pancreatic cancer-derived exosomes taken up by Kupffer cells stimulate secretion of TGFβ and upregulate fibronectin production by hepatic stellate cells, creating a microenvironment allowing for increased recruitment of bone marrow-derived macrophages [61]. One component of cancer-cell derived exosome cargo includes the pro-inflammatory cytokine, migration inhibitory factor (MIF), found to be highly expressed in PDAC-derived exosomes [61]. Furthermore, the blockade of MIF activity prevented liver metastases suggesting the importance of cytokine factors in promoting tumour cells in circulation to adhere to distal sites in the metastatic cascade of disease. 

Another component of tumour-derived exosome cargo, released by colon cancer cells, are miRNAs, which have been demonstrated to support liver metastasis by preparing a pre-metastatic niche [62]. Exosomal miRNA-25-3p derived from circulating tumour exosomes was found to be significantly higher in colon cancer patients with metastatic disease than those without metastasis [60]. The transfer of miRNA-25-3p via exosomes to vascular endothelial cells promotes vascular permeability and angiogenesis in distal organs such as the liver and lung. The up-regulation of expression of pro-angiogenic factors including VEGFR2, ZO-1, occludin, and claudin 5 in endothelial cells allows for the vascularisation required for tumour metastases growth [60]. Exosomes from primary colon tumours carrying miRNA-21-5p specifically accumulate in the liver and induce polarisation of liver macrophages towards an IL-6 producing phenotype by activating TLR-7, thereby creating an inflammatory pre-metastatic environment within the liver and increased metastatic outgrowth [63]. Furthermore, high expression of miRNA-21 in exosomes derived from the plasma of patients with metastatic colon cancer is suggestive of its role in advanced colon cancer [63]. Collectively, these observations elucidate the molecular mechanisms underlying metastatic tropism of colon cancer cells and highlight new possibilities for the prediction as well as prevention of secondary metastasis to the liver. 

### 4.3. Tumour Cell Dormancy—The Sleeping Niche

Tumour cells undergo dormancy post extravasation into the secondary organ due to delayed acclimatisation and hostile stimuli while still maintaining metabolic homeostasis by downregulating oncogenic pathways such as RAS/MAPK and PI3K-AKT signalling [48]. Specific immune cells such has NK cells, CD8+ T cells, IL-1β expressing innate immune cells, and caveolin-1 expressing metastasis associated macrophages are involved in dormancy of cells in the metastatic niche [64]. Dormant cells express weak tumour antigens, thereby escaping immune surveillance and remaining latent until they emerge with increased levels of stemness as aggressive metastases [48]. The pre-metastatic niche also secretes factors that push cells towards dormancy, such as thrombospondin 1 (TSP1) deposited around microvasculature, which blocks tumour angiogenesis, and TGFβ secreted by stromal cells that regulate cancer cell quiescence [55]. Bone morphogenic protein 7 (BMP-7) has been described as another key factor, which activates a metastatic suppressor gene, N-Myc downstream regulated 1 (NDGR1), leading to increased expression of the cell cycle inhibitor p21 and mediating cell cycle arrest to regulate tumour cell dormancy within the metastatic niche [55].

### 4.4. Interactions between Tumour Cells and the Liver Microenvironment

**Figure 2:**
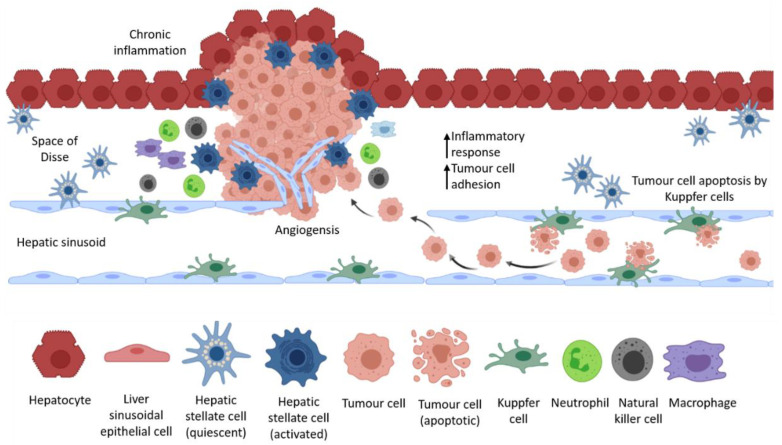
Interactions between liver resident cells and tumour-derived cells in the liver. Circulating tumour cells that become lodged in the hepatic sinusoids evade anti-tumour mechanisms of liver resident cells, form blood vessels that resemble the hepatic sinusoids, and grow as metastatic lesions in the liver.

Circulating tumour cells are mechanically arrested at secondary sites to form lesions, or become stuck in capillaries, growing within the blood vessel before eventual extravasation and colonisation of the secondary organ [48]. After extravasation, tumour cells must develop resistance to immune mechanisms and host tissue defences to survive in the secondary organ in supporting niches [40]. Circulating tumour cells that enter the liver encounter hepatic cell populations and immune cells that are recruited in response to tumour cell invasion (Figure 2) [38]. Since the liver is the site of foetal haematopoiesis, the hepatic environment is capable of regulating self-renewal, proliferation, differentiation, and apoptosis of haematopoietic cells in order to maintain homeostasis of stem cells [65]. During foetal haematopoiesis, haematopoietic stem cells rapidly and extensively proliferate; however, during homeostasis, adult haematopoietic stem cells remain quiescent in the liver and only divide to maintain a normal population of stem cells where one of two daughter cells leaves the niche environment that sustains self-renewal and is exposed to an environment that supports lineage differentiation [66]. The adult liver retains these niche environments, making it a particularly supportive microenvironment for cancer stem cells to rapidly self-renew and differentiate [66,67]. The interaction between circulating tumour cells and the different hepatic cell populations in the liver microenvironment regulates the entry and growth of metastatic tumour cells in the liver. Some of these interactions are outlined in the sections below. 

#### 4.4.1. Liver Sinusoidal Endothelial Cells—The First Line of Defence

Factors secreted by LSECs in response to local ischemia upregulate apoptosis in tumour cells and recruit innate immune cells such as NK cells, which collectively affect the ability of tumour cells to colonise. Although this initial inflammatory response leads to cell death, it can also have a tumour protective effect by upregulating cell adhesion molecules of LSEC cells, therefore increasing cancer cell adhesion and trans-endothelial migration into the space of Disse, thereby evading the cytotoxic effects of KCs and NK cells [38,68,69,70]. LSECs secrete fibronectin that can induce EMT in colon cancer cells by enhancing ERK signalling, and human LSECs were shown to induce cell migration and EMT via MIF, thereby increasing the metastatic potential of colon cancer cells [38,71,72]. Tumour cells that invade between LSECs and the matrix in the space of Disse can form intra-metastatic blood vessels that appear simultaneous with the sinusoidal blood vessels, giving rise to a histologic growth pattern of tumour angiogenesis [38]. The interaction of cancer cells with LSECs initially causes tumour cell destruction in the sinusoids, but can also promote metastasis by enhancing tumour cell migration, invasion, and angiogenesis. 

#### 4.4.2. Diverse Roles of Kupffer Cells in the Formation of a Pre-Metastatic Niche

Kupffer cells (KCs) are involved in promoting the metastatic cascade but can also have a suppressive effect on metastasis depending on the nature of their interaction with invading cancer cells and other immune cells [38]. Tumour cells adhere to KCs in the sinusoidal epithelium, resulting in phagocytosis or apoptosis on entry of circulating tumour cells [38]. On the other hand, KCs can promote extravasation and activation of endothelial cell adhesion molecules (CAMs) if tumour cells survive this initial attack, thereby promoting metastatic colonisation [38]. Upon maintenance of liver homeostasis, KCs show an M2-like macrophage phenotype, which increases expression of anti-inflammatory molecules like IL-10, TNFα, TGFβ, NO, and prostaglandins that inhibit the activation of T cells and promote the proliferation of Tregs [73,74,75]. KCs induce an inflammatory response by shifting towards an M1-like phenotype upon disruption of liver homeostasis by increasing the expression of pro-inflammatory cytokines including IL-6, IL-1β, and TNFα, which recruit neutrophils to the site [73,76], and IL-12 and IL-18, which recruit NK cells that produce IFNγ [73]. KCs also secrete hepatocyte growth factor (HGF), VEGF, and matrix metalloproteinases (MMPs) such as MMP-9 and MMP14 that promote tumour cell invasion in the parenchymal space as well as tumour cell proliferation and angiogenesis [38]. 

#### 4.4.3. Hepatic Stellate Cells Function as Cancer-Associated Fibroblasts 

Hepatic stellate cells (HSCs) are liver resident mesenchymal cells that retain features of resident fibroblasts when embedded in normal stromal matrix but of pericytes when attached to endothelial cells of capillaries [77]. They are normally found quiescent in the space of Disse, activated in response to tissue injury, and are responsible liver fibrosis [38,77]. Once activated, they acquire a myofibroblast phenotype and produce ECM rich in collagen I and IV, leading to cirrhosis [38]. They also secrete endothelin-1, which is a vasoconstrictor that promotes cell proliferation, fibrogenesis, and contraction linked to cirrhosis [77]. Chemokines and cytokines released by these cells also recruit inflammatory and immune cells, thereby modulating the hepatic immune environment [78,79]. Liver-resident HSCs have been previously shown to promote tumour cell proliferation and invasion in hepatocellular carcinoma [80,81]. It has also been shown that HSCs exert immunosuppressive effects by inhibiting T cell proliferation and inducing apoptosis in activated T cells [78,82,83,84,85]. HSCs promote Treg cell expansion by inducing T cell hypo-responsiveness, a state in which T cells are unresponsive to further stimuli, thereby allowing hepatocellular carcinoma cells to escape immune surveillance and develop as tumours in the liver [78]. Furthermore, HSCs promoted cell proliferation and migration in vitro, and the presence of HSCs increased the number of liver metastases and increased metastatic cell proliferation in vivo [78]. This tumour promoting effect is mediated by the HSCs via the secretion of TGFβ [32]. The CXCR4/TGFβ signalling axis has been recently implicated in colon cancer metastasis to the liver, as CXCR4 and CAF markers such as α-SMA, vimentin, FSP1, and FAP were highly expressed in patient specimens of liver metastatic lesions [86]. TGFβ induces the differentiation of HSCs into CAFs, thereby promoting CRC liver metastasis both in vitro and in vivo [86]. Moreover, CXCR4/TGFβ blockade by AMD3100 inhibited the differentiation of HSCs to CAFs and significantly reduced metastatic burden in vivo [86]. These observations indicate that therapeutically targeting HSCs to inhibit secondary CRC metastasis to the liver could be beneficial. 

## 5. Heterogeneity of the Tumour Immune Microenvironment Dictates Therapeutic Responses 

### 5.1. Patterns of Tumour Immunogenicity and Cytokine Profiles 

The characterisation of tumour heterogeneity defined by the differences in tumour cell genomic signatures, varied immune cell infiltration, and states of cell differentiation will in future allow for tailored treatments that specifically address the varied disease context. Correlation of mutational burden, neoantigen load, and presence of known oncogenic mutational signatures with the spatial distribution and activation/differentiation status of immune cells is an important next step in identifying cancer treatments. The heterogeneous nature of primary colon tumours and their immunogenicity is described via three distinct patterns [87]. “Hot tumours” describe those where the cytotoxic lymphocytes (CTLs) are infiltrated and inflamed, showing high expression of activation marks such as PD1 and PDL1, with increased IFNγ and granzyme B, and a low stromal component (CMS1 subtype). These are likely to be MSI-high and good candidates for current immunotherapy treatments. Tumours in which current immune checkpoint blockade would likely fail include the “CTL infiltrated, excluded” tumours, in which the dense structure generated by ECM-rich CAFs house tumour-associated macrophages to keep CTLs trapped in the periphery of the lesions. The third immune-subtype is associated with the presence of tertiary lymphoid structures (TLS), which provide a ‘direct source’ of Tregs, B cells, and dendritic cells [87]. 

Krummel and colleagues demonstrate emerging evidence linking the mutational status and activation of cell signalling cascades intrinsic to a tumour cell to specific cytokine activity [87]. For example, the *KRAS G12D* mutation is associated with increased GM-CSF [88,89] and LIF levels [90,91] that contribute to increased influx of CD11b^+^ myeloid cells [87,92,93]. In melanoma, the *BRAF V600E* mutation is linked to increased STAT3 activity and increased expression of IL-1, IL-10, and IL-6, which in turn influence dendritic cell activity within the tumour microenvironment [87,94]. 

Experimentally, this concept is demonstrated in elegant animal experiments in which mice with combined gene deletions in *Apc*, *Trp53*, *Tgfbr2,* and the *KrasG12D* mutation led to the development of metastatic colon cancer, with similar features to human tumours that possess the immune-excluded or CMS4 subtype [33]. Interestingly, these tumours induced the over-production of TGFβ in the CAFs and treatment with galunisertib, the TGFBR1 specific inhibitor to block CAF: TGFβ responses, reduced tumour growth, and metastasis. Given the effects of galunisertib on metastasis in transplanted mutant organoids, one can conclude that the anti-metastatic effects of galunisertib would also block TGFβ signalling in the metastatic niche including metastatic outgrowth in the liver [33]. Interestingly, the effects of galunisertib also included increased infiltration of CD4^+^ T cells and of T-bet^+^ lymphocytes, in addition to increased PD-1 expression on lymphocytes and macrophages. Accordingly, the co-treatment of galunisertib with anti-PD-L1 treatment induced an immune response characterised with elevated T-bet and IFNγ levels in CD4^+^ T cells, increased GZMB production, along with increased infiltration into the tumours. As a result, a profound effect on metastasis was observed attributing immune cell evasion as a key mechanism for metastatic outgrowth. In line with these observations, targeted treatments that modulate specific cytokine signalling pathways may sensitise colon tumours to checkpoint inhibition.

### 5.2. Targeted Treatments for Metastatic Colon Cancer 

Monoclonal antibodies that target the epidermal growth factor receptor (EGFR), cetuximab and panitumumab, were the first approved targeted treatments for CRC and have been hugely successful treatments in patients. Cetuximab in combination with fluorouracil, leucovorin, and irinotecan (FOLFIRI) allows for improved benefits in patients with primary tumours as well as metastatic disease [95,96]. This combination treatment is particularly effective in enhancing mCRC patient survival if patients do not have RAS mutations [96].

Bevacizumab is also an FDA-approved targeted therapy, a monoclonal antibody against vascular endothelial growth factor (VEGF) directed primarily at blocking tumour angiogenesis but also with effects directly on tumour cells. Bevacizumab is used as standard first-line regimen in combination with 5FU-based chemotherapy in patients with mCRC. Among chemotherapy regiments, a combination of fluorouracil, leucovorin, oxaliplatin, and irinotecan (FOLFOXIRI) has shown superior efficacy as compared with FOLFIRI. The combination of FOLFOXIRI with bevacizumab is shown to be more effective compared with FOLFIRI and bevacizumab in reducing disease in patients with metastatic colorectal cancer [97,98] (Table 2). Interestingly, it has been reported that mCRC patients with right-sided lesions, who normally present with worse disease outcomes compared to those with left-sided tumours, have shown improved responses to combined FOLFOXIRI and bevacizumab treatment irrespective of BRAF and RAS mutational status [98].

Other targeted therapies for VEGFRs have been developed and approved for use in mCRC patients, such as aflibercept, which is a recombinant fusion protein that combines sequences to the extracellular domain of VEGFR with the Fc region of human immunoglobulin G_1_ (IgG_1_). Functioning as a soluble receptor, aflibercept competes for ligand binding with the two forms of VEGFR (A and B) as well as placental growth factor, showing efficacy in progression free survival, as a combination treatment with FOLFOXIRI [99,100]. Ranibizumab, a humanised, monoclonal antibody that neutralises all forms of vascular endothelial growth factor (VEGF) A, is also as treatment in mCRC irrespective of KRAS mutation status [101,102]. A comparison of clinical data from randomised studies of anti-EGFR and anti-VEGF agents in advanced, metastatic colon cancer indicate better outcomes in objective response rates as well as overall survival with first-line anti-EGFR therapy compared with anti-VEGF therapy irrespective of KRAS or RAS mutational status [103]. 

Small molecule inhibitors targeting receptor tyrosine kinases (RTKs) is a strategy that has led to the development of a range of drugs that have been approved for use in solid cancers. However, regorafenib is currently the only multi-kinase inhibitor that has been approved by the FDA as treatment of mCRC. Regorafenib has inhibitory effects on VEGFR1-3, PDGFR and FGFR, KIT, RET, RAF-1, and BRAF, thereby modulating proliferation in addition to angiogenesis, and has been shown to improve survival in patients with treatment-refractory mCRC [104,105]. Regorafenib is still under clinical trials in combinations with cetuximab or panitumumab, in patients with unresectable, locally advanced, or metastatic colorectal cancer (NCT04117945, NCT03844620), or in combination with pembrolizumab, ipilimumab, and nivolumab (NCT03657641, NCT04362839) (Table 2). One significant treatment-emergent adverse event is hypertension [106], and its management with anti-hypertensive medication targeting the renin-angiotensin system may have added positive anti-metastatic effects [107].

Emerging discoveries with small molecule inhibitors continue to show promise as tailored treatments that address the heterogeneous nature of colon cancer. Small molecule inhibitors such as MRTX849 have been identified as potent, selective KRAS G12C inhibitors to selectively modify mutant cysteine 12 in the GDP-bound KRAS G12C mutant protein to inhibit signalling [108]. MRTX849 demonstrated pronounced tumour regression and objective responses in patients with KRAS G12C positive colon adenocarcinomas. Clinical improvement of reduction in tumour burden by 47% was observed in a female patient with advanced metastatic spread to the liver, peritoneum, ovaries, and lymph nodes treated with MRTX849 [109]. Currently trials are underway to evaluate clinical responses in a range of cancers (NCT03785249).

### 5.3. Role of Immunotherapies in the Treatment of Metastatic Colon Cancer 

Metastatic colorectal cancer harbouring genetic defects in the mismatch-repair pathway presents with a high tumour mutational burden and is highly sensitive to anti–PD-1 antibody therapy. In a recently reported phase III study of 307 patients with metastatic microsatellite instability high, deficient mismatch repair (MSI-H–dMMR) colorectal cancer, in which pembrolizumab activity was compared with 5-fluorouracil–based chemotherapy with or without bevacizumab or cetuximab, pembrolizumab was found to be superior to chemotherapy with respect to progression-free survival with an overall complete or partial response observed in 43.8% of the patients in the pembrolizumab group and 33.1% in the chemotherapy group, while treatment-related adverse events were significantly lower in the pembrolizumab cohort compared to those receiving chemotherapy [110]. While longer term patient outcomes of the clinical trials are still being monitored, pembrolizumab treatment resulted in significantly longer progression-free survival than chemotherapy when received as first-line therapy for MSI-H–dMMR mCRC, with fewer treatment-related adverse events (NCT02563002) [110]. The findings of this study had led to the FDA, in June 2020, approving pembrolizumab as a first-line treatment for patients with MSI-H-dMMR mCRC. The impact of immune checkpoint inhibitors used in conjunction with other targeted therapies presents a new frontier for the management of advanced mCRC and may have therapeutic potential in treatment refractory mCRC.

## 6. Summary 

To date, the treatment of advanced mCRC has involved chemotherapy combined with a range of targeted therapies. These combinations have been successful and resulted in improved management of disease and better overall survival outcomes. However, critical issues that still impair patient quality of life include treatment resistance, recurrent disease, and distal metastasis. The use of molecular classification of colon cancer subtypes, driver mutation characterisation, and MMR-status now influences treatment decisions. Data obtained regarding treatment efficacy on clinical outcomes in stratified patient groups will assist in further progress towards individualised treatments for patients. The development of preclinical models of the different colon cancer subtypes, as well as an improved understanding of the interplay between tumour lesions within the metastatic niche, will greatly advance the identification of novel targets for drug development. 

## Figures and Tables

**Table 1:** CRC consensus molecular subtypes.

	CMS1 MSI Immune	CMS2 Canonical	CMS3 Metabolic	CMS4 Mesenchymal
**~% CRC Cases**	14%	37%	13%	23%
**Key Characteristics**	MSI High	MSI low	Mixed MSI	MSI low
	Somatic copy number alteration (SNCA) low	SNCA high	SNCA low	SNCA high
	Hypermethylation		Hypermethylation	
	Significant immune infiltration			Stromal infiltration
**Altered Pathways**	DNA mismatch repair (PD1, PD-L1)	Wnt	Metabolic pathways	TGFβ
	Jak/STAT	Myc		EMT
				ECMremodeling
**Key Gene Mutations**	BRAF	TP53	KRAS	
			PIC3CA	

Molecular classification of colon cancer [20,24,25].

**Table 2:** Targeted treatment regimens for mCRC.

Molecular Target	Targeted Therapy	Approved or Trial Reference	Treatment Regimens
EGFR	Cetuximab	Approved	FOLFIRI + Cetuximab
	Cetuximab	NCT01228734	*FOLFOX *FOLFOX + Cetuximab
	Cetuximab	EPIC	*Irinotecan *Irinotecan + Cetuximab
	Panitumumab	NCT00364013	*FOLFOX *FOLFOX + Panitumumab
	Panitumumab	NCT00339183	*FOLFIRI *FOLFIRI + Panitumumab
	Panitumumab	NCT03231722	*FOLFOX +Cetuximab *FOLFIRI + Panitumumab
VEGF	Bevacizumab	Approved	FOLFIRI + Bevacizumab
	Bevacizumab	NCT00719797	*FOLFOX +Bevacizumab *FOLFIRI + Bevacizumab
	Bevacizumab	NCT00484939	*Capecitabine *Bevacizumab + Capecitabine
	Afibercept	NCT01661270	*FOLFIRI *FOLFIRI + Afibercept
Receptor Tyrosine Kinase and KRAS	Regorafenib	NCT 04117945	*Regorafenib + Cetuximab/Panitumumab * Cetuximab/ Panitumumab + Regorafenib
	Regorafenib	NCT03844620	CTCs as an indicator of treatment response to Regorafenib
	Regorafenib	NCT03657641	Regorafenib + Pembrolizumab
	Regorafenib	NCT04362839	Regorafenib + Ipilimumab + Nivolumab
	MRTX849	NCT03785249	*MRTX849 *MRTX849 + Cetuximab *MRTX849 + Pembrolizumab *MTRX849 + Afatinib

A summary of targeted treatment regimens approved or in clinical trials for mCRC. This summary is not exhaustive, however provides an overview of some of the key treatment regimens available in the clinic.

## Data Availability

Data sharing is not applicable to this article.
